# miR-124 Intensified Oxaliplatin-Based Chemotherapy by Targeting CAPN2 in Colorectal Cancer

**DOI:** 10.1016/j.omto.2020.04.003

**Published:** 2020-04-14

**Authors:** Xu-Qin Xie, Mo-Jin Wang, Yuan Li, Lin-Ping Lei, Ning Wang, Zhao-Ying Lv, Ke-Ling Chen, Bin Zhou, Jie Ping, Zong-Guang Zhou, Xiao-Feng Sun

**Affiliations:** 1 Institute of Digestive Surgery, State Key Laboratory of Biotherapy and Cancer Center, West China Hospital, Sichuan University, Chengdu 610041, Sichuan Province, China; 2 Department of Gastrointestinal Surgery, West China Hospital, Sichuan University, 37 Guo Xue Xiang, Chengdu 610041, Sichuan Province, China; 3 The First Affiliated Hospital of Dali University, 32 Jiashibo Avenue, Dali 671000, Yunnan Province, China; 4 Division of Epidemiology, Vanderbilt University Medical Center, 2525 West End Avenue, Suite 800, Nashville, TN 37203-1738, USA; 5 Department of Oncology and Department of Biomedical and Clinical Sciences, Linköping University, 581 83 Linköping, Sweden

**Keywords:** colorectal cancer, miR-124, CAPN2, oxaliplatin, chemotherapy

## Abstract

Our previous study demonstrated that miR-124 was downregulated in colorectal cancer (CRC) compared with normal mucosa, and the downregulated expression of miR-124 was an independent prognostic factor in CRC patients. However, the function of miR-124 in CRC patients treated with chemotherapy is currently unclear. The aim of this study was to determine the miR-124 expression and its regulative role in oxaliplatin (L-OHP)-based chemotherapy of CRC patients. We observed that low miR-124 expression was correlated with worse overall survival (OS) in the 220 patients who received postoperative chemotherapy of 5-fluorouracil [5-FU]+leucovorin+L-OHP (FOLFOX) or capecitabine+L-OHP (XELOX). miR-124 overexpression promoted L-OHP-induced, but not 5-FU-induced, cytotoxicity and apoptosis in HT29 and SW480 cells. CAPN2 was a direct target of miR-124, and its protein expression was reduced by forced expression of miR-124. miR-124 inhibited tumorigenesis and promoted OS of mice bearing xenograft tumors, especially upon L-OHP treatment. miR-124 also promoted L-OHP-induced apoptosis and restrained CAPN2 protein expression in xenograft tumors. Our results suggest that miR-124 could be considered as both a predictor of L-OHP-based chemotherapy for personalized treatment and a therapeutic target for CRC.

## Graphical Abstract

**Figure:**
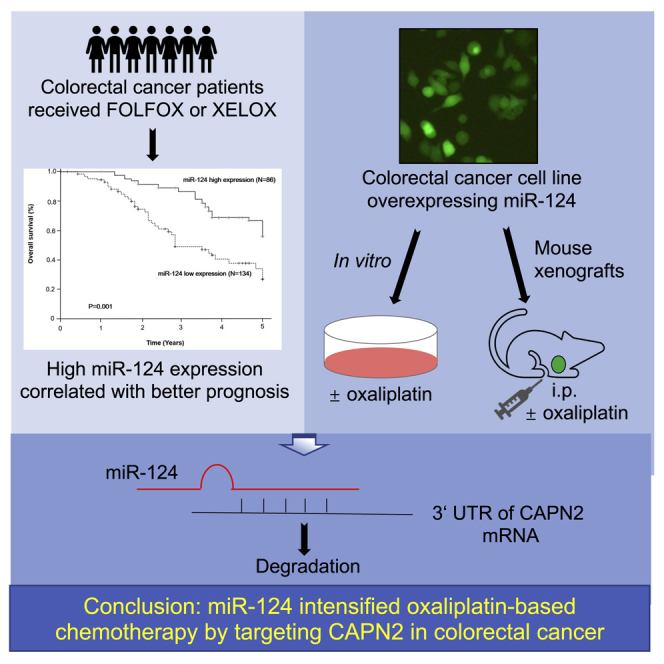

## Teaser

Sun and colleagues determined miR-124 expression and its regulative role in oxaliplatin-based chemotherapy in colorectal cancer patients, and the evidence was also verified in cancer cell lines and a mouse model. Mechanistically, miR-124 functioned by directly interacting with the 3′ UTR mRNA of CAPN2, a calcium-dependent protease.

## Introduction

Colorectal cancer (CRC) has been a serious public health problem worldwide for many years because of its frequency, despite the screening and preventive strategies. Despite recent advances in surgery technique and comprehensive treatment, CRC remains associated with a poor prognosis, especially with very low rates of long-term survival among patients with advanced disease.^1^^,^^2^ Therefore, accurate determination of the prognosis is crucial for practitioners in order to optimize and personalize treatment strategies. Given the poor prognosis of CRC patients, it is essential to validate new prognostic markers.

MicroRNAs (miRNAs) belong to a group of small non-coding RNAs of 18–24 nt that are known to significantly regulate gene expression, posttranslational repression, or cleavage of mRNA targets.^3^ Most miRNAs behave as oncogenes or tumor suppressor genes, depending on the target mRNA, and they play a role in the pathogenicity of various malignancies.^4^ More and more molecular studies indicate a closer link between miRNA functions and CRC pathogenesis, including oncogene activation and anti-oncogene inactivation, influencing proliferation and apoptosis, and regulation of pathogenesis. Investigation of these miRNAs would expand our view to better understand CRC carcinogenesis.

miR-124, a brain-enriched miRNA emerging as an important regulator of gastrulation and neural development, has recently been confirmed as a tumor suppressor through targeting several crucial genes, such as Slug, LHX2, and CBL.^5^, ^6^, ^7^ As a potential tumor suppressor gene, miR-124 has been shown to regulate proliferation, migration, and invasion in certain cancers.^8^, ^9^, ^10^, ^11^ Pertinent to chemotherapy, Qiao et al.^12^ reported that miR-124 could enhance sensitivity to temozolomide (TMZ) by downregulating aurora kinase A (AURKA) in glioblastoma cancer. Furthermore, the study of Wang et al.^13^ showed that sulforaphane-induced miR-124 upregulation sensitized gastric carcinoma cells to cisplatin via the miR-124/interleukin (IL)-6R/ signal transducer and activator of transcription 3 (STAT3) axis. Our previous study showed that miR-124 expression was significantly downregulated in CRC compared to normal mucosa, and downregulated miR-124 expression correlated significantly with worse prognosis.^14^ However, in our previous study we did not analyze the relationship between the expression of miR-124 and chemotherapy for diverse regimens. As we known, chemotherapy is a universally acclaimed effective primary treatment in advanced CRC.

FOLFOX (5-fluorouracil [5-FU]+leucovorin+oxaliplatin [L-OHP]) and XELOX (capecitabine+L-OHP) are still the first-line options for treating advanced CRC. The main active ingredients of both FOLFOX and XELOX are 5-FU and L-OHP. 5-FU is a potent antitumor agent and is metabolized to ribonucleotides and deoxyribonucleotides, which can be incorporated into RNA and DNA. Through its incorporation, 5-FU exerts cytotoxic effects on tumor cells through thymidylate synthase inhibition and modification of RNA. 5-FU has been widely prescribed for solid tumors since it was first introduced by Heidelberger et al.^15^ in 1957. L-OHP belongs to the family of platinum, analogous to carboplatin and cisplatin, and has been known for several years for its chemotherapeutic compound. DNA damage is largely responsible for the cytotoxic properties of platinum (Pt) complexes.^16^ L-OHP is particularly effective in the treatment of primary advanced CRC.^17^ Despite the widespread clinical use of 5-FU and L-OHP, the molecular mechanisms behind their cytotoxic effects have not been completely understood. Furthermore, clinical prognostic markers of response to 5-FU and L-OHP remain poorly characterized. Therefore, we determined the role of miR-124 in the prediction and modulation of cellular responses to 5-FU- and L-OHP-based chemotherapy in this study. We also studied the possible targets and intracellular pathways involved in these processes.

Calpain (CAPN) proteins are calcium-dependent proteases and are implicated in a wide range of molecular, cellular, and physiological functions.^18^ Of the 14 known mammalian isoforms, the ubiquitously expressed CAPN1 protein (μ-calpain) and CAPN2 protein (m-calpain) have been studied most extensively. Lakshmikuttyamma et al.^19^ have reported that the activity and protein expression of CAPN2 were significantly higher in CRC than in normal colonic mucosa. Rose et al.^20^ found that CAPN2 protein inhibitor therapy reduces murine colitis and colitis-associated cancer. Pertinent to chemotherapy, Storr et al.^21^ have reported that high expression of CAPN2 protein was significantly associated with resistance to platinum-based adjuvant chemotherapy in ovarian cancer. Using TargetScan, we found that CAPN2 is predicted to be a target of miR-124. Therefore, we suspected that miR-124 may regulate chemosensitivity in CRC by inhibiting CAPN2.

In the present study, we investigated the association between miR-124 expression and prognosis in stage II/III CRC patients who received postoperative FOLFOX or XELOX chemotherapy. Furthermore, miR-124-overexpressing cell lines and a xenograft animal model were established in order to explore the role of miR-124 in the L-OHP-based chemotherapy response. We also investigated the association between CAPN2 and miR-124 using a luciferase reporter assay and western blot analysis.

## Results

### Relationship between Expression of miR-124 and Prognosis of CRC Patients Who Received FOLFOX or XELOX

**Figure 1:**
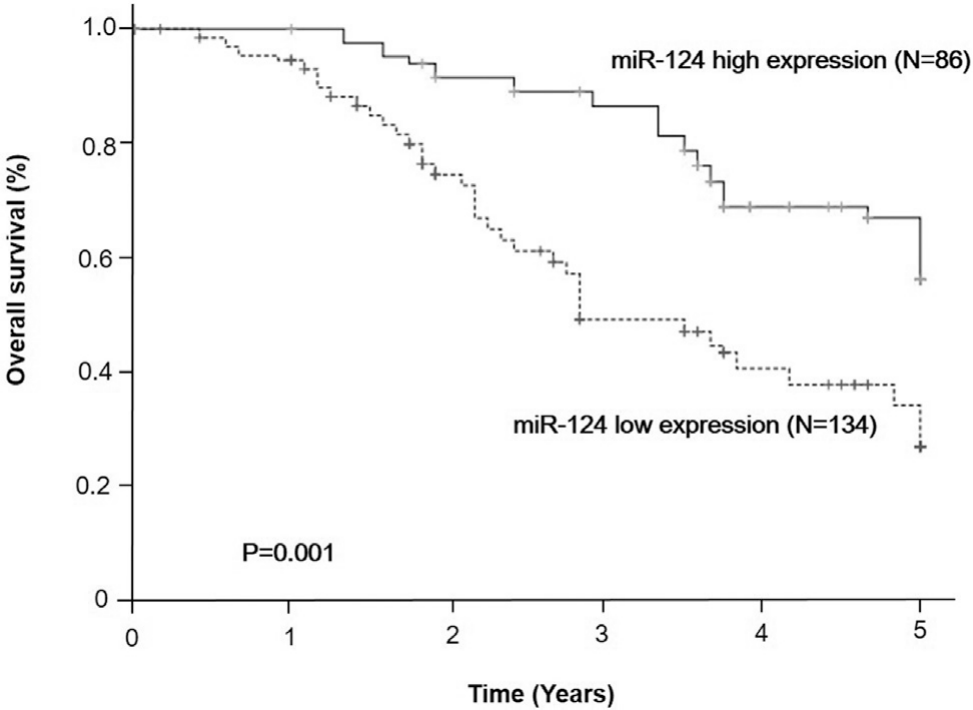
Higher miR-124 Expression Predicted Better Overall Survival in CRC Patients Who Received FOLFOX or XELOX The Kaplan-Meier plotter presented the overall survival according to miR-124 status (high expression, N = 86; low expression, N = 134). FOLFOX, fluorouracil+oxaliplatin; XELOX, capecitabine+oxaliplatin.

We assessed miR-124 expression levels in 220 tumors with the matched normal mucosa samples from CRC patients who received FOLFOX or XELOX. Univariate survival analysis showed that the group with low miR-124 expression had worse overall survival (OS) than did the high miR-124 expression group (p = 0.001, Figure 1). Five-year OS was 56.2% in the high miR-124 expression group but only 27.0% in the low miR-124 expression group. In further multivariate regression analyses, the prognostic significance still remained after adjusting for patients’ sex, age, tumor location, TNM (tumor, node, metastasis) stage, differentiation, and histological type (hazard ratio [HR], 2.279; 95% confidence interval [CI], 1.468–3.538; p < 0.001, Table 1) of CRC patients who received FOLFOX or XELOX.

**Table 1:** Multivariate Survival Analysis of miR-124 Expression, Sex, Age, Stage, Grade of Differentiation, and Histological Type in Relationship to Overall Survival in Colorectal Cancer Patients Who Received FOLFOX (fluorouracil+oxaliplatin) or XELOX (capecitabine+oxaliplatin) Therapy

Variable	Unfavorable/Favorable	HR	95% CI	p Value
miR-124 expression	downregulated/upregulated	2.279	1.468–3.538	<0.001
Sex	male/female	0.884	0.575–1.361	0.576
Age	>66/≤66 years	1.880	1.228–2.880	0.004
Stage	III/II	2.394	1.495–3.833	<0.001
Differentiation	poor/moderate+well	1.290	0.857-1.942	0.223
Histological type	mucinous/nonmucinous	0.855	0.495–1.475	0.573

HR, hazard ratio; CI, confidence interval.

### The Effect of miR-124 Overexpression on Chemosensitivity in CRC Cells and Underlying Mechanism

**Figure 2:**
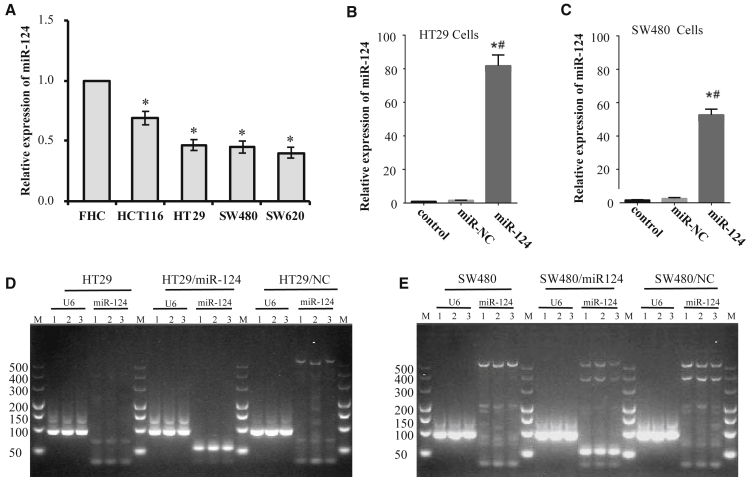
miR-124 Stable Overexpressing Cell Lines Were Established on HT29 and SW480 Cells miR-124 expression was analyzed by quantitative real-time PCR, and U6 expression was used as an internal control. Data are presented as means ± SD from three independent experiments with triple replicates per experiment. (A) miR-124 expression in FHC cells and four human CRC cell lines was analyzed by quantitative real-time PCR and normalized to the value of FHC cells. ∗p < 0.05 compared to FHC. (B and C) Relative miR-124 expression in HT29 cells (B) or SW480 cells (C) stably transfected with/without miR-124 or miR-NC was determined and normalized to the value of control cells. ∗p < 0.05 compared to control; ^#^p < 0.05 compared to miR-NC. (D and E) Quantitative real-time PCR products from HT29 cells (D) or SW480 cells (E) transfected with/without miR-124 or miR-NC were analyzed by agarose gel electrophoresis. The molecular mass of the miR-124 band is 70 kb, and the U6 band is 94 kb.

The expression levels of miR-124 were compared in human colon epithelial cell lines of fetal cells and four CRC cell lines, including HCT116, HT29, SW480, and SW620, by using quantitative real-time PCR. As shown in Figure 2A, miR-124 expression levels were higher in FHC cells than those in the four CRC cell lines (p < 0.05, CRC versus FHC). We suspected that restoration of miR-124 expression levels might enhance the anti-cancer efficacy of chemotherapy in CRC. A quantitative real-time PCR assay revealed that miR-124 expression levels in HT29/miR-124 cells (p < 0.001, Figure 2B) and SW480/miR-124 cells (p < 0.001, Figure 2C) were much higher than those in their parental cells. Furthermore, quantitative real-time PCR products obtained from these cell lines were validated by using agarose gel electrophoresis. As expected, obvious miR-124 bands were only observed in quantitative real-time PCR products from HT29/miR-124 cells and SW480/miR-124 cells (Figures 2D and 2E).

**Figure 3:**
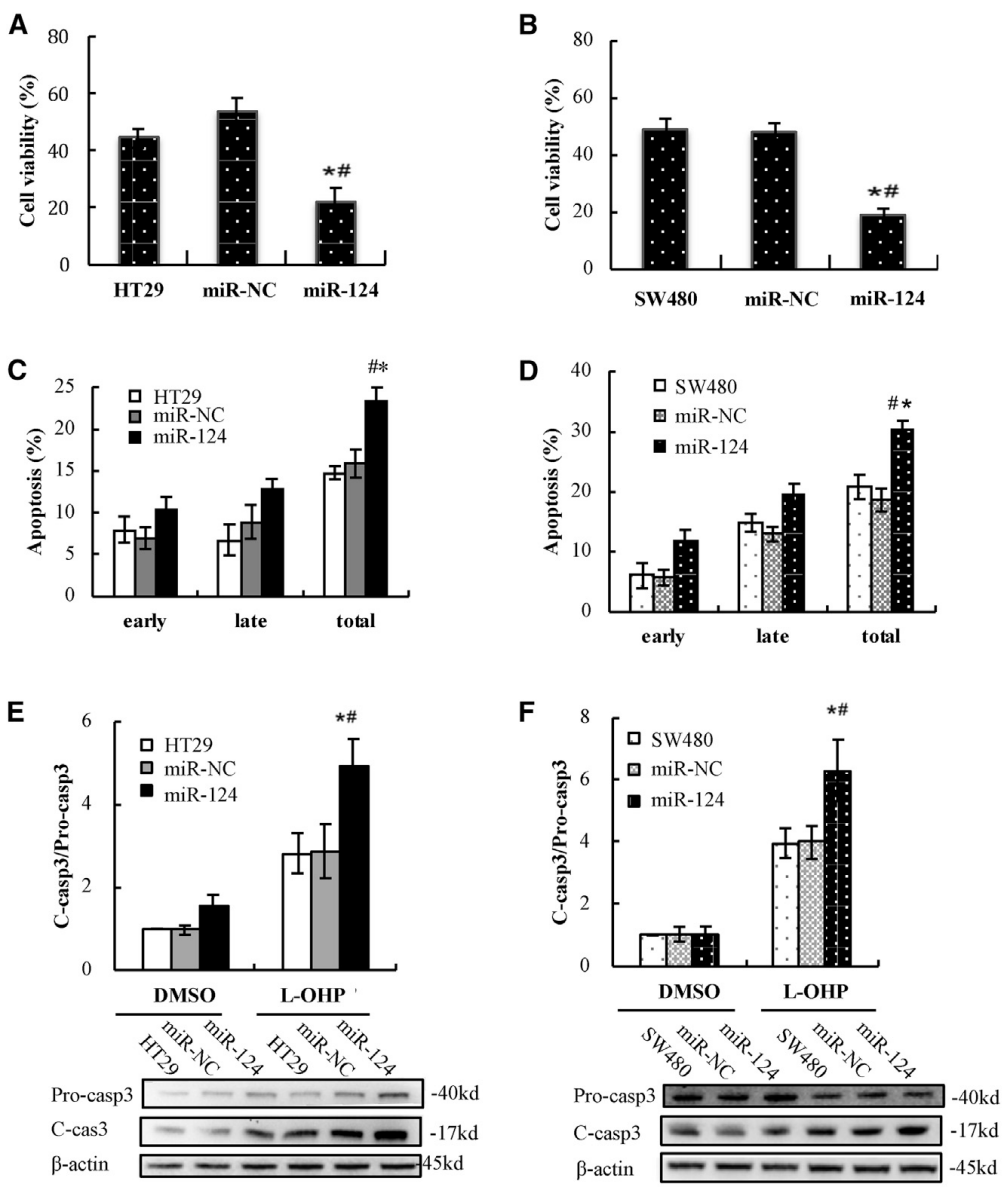
L-OHP-Induced Cytotoxicity and Apoptosis Were Enhanced by miR-124 Overexpression in HT29 and SW480 Cells (A and B) Cytotoxic effects of L-OHP on HT29 cells (A) or SW480 cells (B) stably transfected with/without miR-124 or miR-NC were detected by a CCK-8 assay. (C and D) L-OHP-induced apoptotic cells in HT29 cells (C) or SW480 cells (D) stably transfected with miR-124 or miR-NC were determined by flow cytometry and analyzed with a histogram. (E and F) Proteins of active caspase-3 and pro-caspase-3 in HT29 cells (E) or SW480 cells (F) with/without transfection with miR-124 or miR-NC were detected by western blot after treatment with L-OHP or vehicle (0.1% DMSO), and the ratios of active caspase-3 to pro-caspase-3 were analyzed in a histogram. Data are presented as means ± SD of three independent experiments with triple replicates per experiment. ∗p < 0.05 versus control. ^#^p < 0.05 versus miR-NC.

Cytotoxicity studies were carried out by using a Cell Counting Kit-8 (CCK-8) assay, representing the percentage of viability inhibition induced by treatments. As we known, the main active ingredients of both FOLFOX and XELOX are 5-FU and L-OHP. We wondered whether miR-124 affected chemosensitivity of CRC cells to 5-FU, L-OHP, or both. When HT29, HT29/negative control (NC), and HT29/miR-124 cells were treated with 5 μM 5-FU for 72 h, their viability did not show significant differences (HT29/miR-124 versus HT29, p = 0.524; HT29/miR-124 versus HT29/NC, p = 0.827, Figure S1A). The viability of SW480, SW480/NC, and SW480/miR-124 also did not show marked differences after 5-FU treatment for 72 h (SW480/miR124 versus SW480, p = 0.527; SW480/miR124 versus SW480/NC, p = 0.547, Figure S1B). These results suggested that miR-124 overexpression did not change chemosensitivity to 5-FU in the cells. After treatment with 5 μM L-OHP for 72 h, viability of HT29/miR-124 was significantly downregulated compared to HT29 and HT29/NC cells (HT29/miR124 versus HT29, p = 0.007; HT29/miR-124 versus HT29/NC, p = 0.002, Figure 3A). Similarly, miR-124 significantly improved L-OHP-induced cytotoxicity in SW480 cells (SW480/miR124 versus SW480, p = 0.002; SW480/miR-124 versus SW480/NC, p = 0.001, Figure 3B). These results indicated that miR-124 overexpression promoted chemosensitivity to L-OHP but not to 5-FU in CRC cells. Therefore, we only determined the regulating effect of miR-124 on chemosensitivity to L-OHP and the underlying mechanism in the following experiments.

We asked whether miR-124 overexpression aggravated cytotoxicity of L-OHP through promoting apoptotic cell death in CRC cells. To address the issue, HT29, HT29/NC, and HT29/miR-124 cells were treated with 5 μM L-OHP for 72 h. Apoptotic death cells were quantitatively detected by a 7-aminoactinomycin D (7-AAD)/allophycocyanin (APC) binding assay. The apoptosis rate was significantly increased in HT29/miR-124 cells compared to HT29 and HT29/NC cells after L-OHP treatment (HT29/miR124 versus HT29, p < 0.001; HT29/miR124 versus HT29/NC, p = 0.001, Figure 3C). Similarly, L-OHP-induced apoptosis also markedly increased in SW480/miR-124 cells compared to SW480 and SW480/NC cells (SW480/miR124 versus SW480, p = 0.001; SW480/miR124 versus SW480/NC, p < 0.001, Figure 3D).

To provide biochemical evidence for the occurrence of apoptosis, we examined whether miR-124 overexpression increased the expression level of active caspase-3 upon L-OHP treatment. As expected, L-OHP treatment markedly increased the ratio of cleaved caspase-3/pro-caspase-3 in HT29/miR-124 and SW480/miR-124 cells compared with their parental cells (Figures 3E and 3F). Although the value of cleaved caspase-3/pro-caspase-3 did not significantly changed in miR-124-overexpressing CRC cells compared with their parental cells after vehicle (0.1% DMSO) treatment, both cleaved caspase-3 and pro-caspase-3 expression slightly increased after miR-124 overexpression.

**Figure 4:**
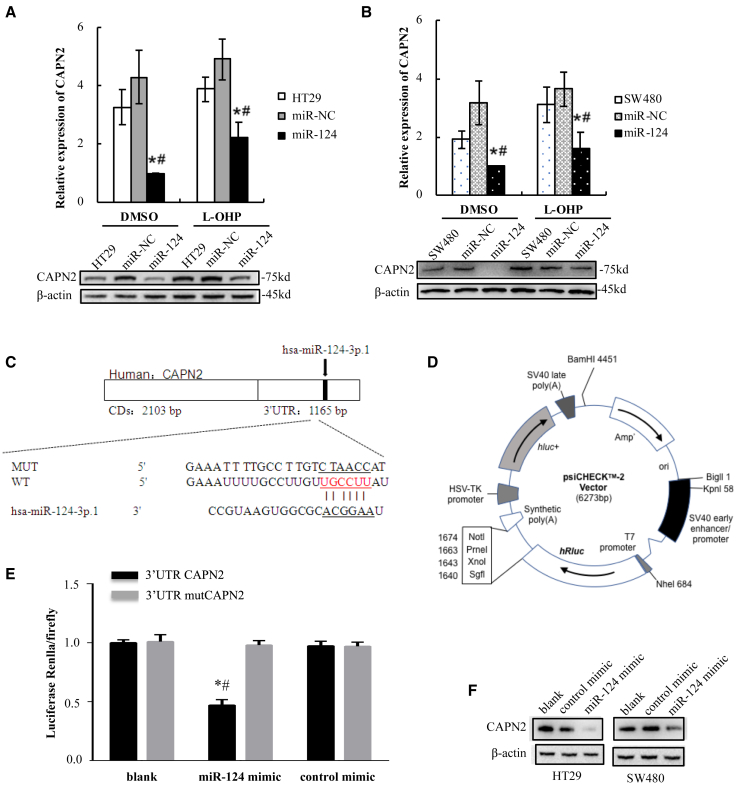
miR-124 Directly Targeted the 3′ UTR of CAPN2 and Suppressed CAPN2 Protein Expression (A and B) CAPN2 protein expression in HT29 cells (A) and SW480 cells (B) treated with L-OHP or vehicle (0.1% DMSO) was detected by western blot and analyzed with a histogram. Data are presented as the means ± SD from three independent experiments with triple replicates per experiment. ∗p < 0.05 versus control; ^#^p < 0.05 versus miR-NC. (C) Nucleotides between the miR-124 total sequence and the target CAPN2 3′ UTR sites were compared. The link lines connect two paired nucleotides. The red ones represent the shared region with which miR-124. The wild-type (WT) CAPN2 3′ UTR and the mutational 3′ UTR were inserted into Dual-Luciferase reporter plasmid. (D) Plasmid profiles of Dual-Luciferase reporter included CAPN2 3′ UTR WT/mut fragments. (E) A luciferase reporter assay was performed using HEK293T cells to detect the relative luciferase activities of WT and mut CAPN2 reporters. The Renilla luciferase vector was used as an internal control. (F) Protein expression levels of CAPN2 and β-actin were determined in HT29 cells (left bands) or SW480 cells (right bands) after transfection with miR-124 mimic or control mimic using western blot analysis. Data are presented as the means ± SD from three independent experiments with triple replicates per experiment. ∗p < 0.05 versus blank; ^#^p < 0.05 versus control mimic.

CAPN2 is predicted to be a target of miR-124 when using TargetScan. We found that overexpression of miR-124 resulted in a sharp decrease in CAPN2 protein expression in HT29 cells (Figure 4A) and SW480 cells (Figure 4B) treated with L-OHP or vehicle. This suggested that CAPN2 might be a target of miR-124 and play a role in regulating chemosensitivity to L-OHP. In order to analyze whether the decrease in CAPN2 protein expression was due to the direct interaction of miR-124 with the 3′ UTR of CAPN2, luciferase assays were performed. First, TargetScan was used to identify potential miR-124 targets. We found that the nucleotide 926–932 region within the 3′ UTR of the CAPN2 gene contained a candidate binding site for miR-124 by using TargetScan (Figure 4C). Then, we designed the mutated target sequence and constructed the wild-type (WT) and mutation-type (mut) reporter gene plasmid vectors (Figure 4D), and co-transfected them along with miR-124 mimics or controls into human embryonic kidney 293T (HEK293T) cell lines. The luciferase assays showed that HEK293T cells co-transfected with pGL3-Capn2-WT and miR-124 mimics expressed significantly decreased luciferase activities compared to the cells co-transfected with pGL3-Capn2-mut and miR-124 mimics or pGL3-Capn2-WT and scrambled miRNA controls (3′ UTR CAPN2+hsa-miR-124 versus 3′ UTR CAPN2, p = 0.018; 3′ UTR CAPN2+hsa-miR-124 versus 3′ UTR CAPN2+NC, p = 0.040, Figure 4E). However, the mimics did not work on the mutated target. These results demonstrated that miR-124 directly targeted the 3′ UTR of CAPN2. To further confirm the effect of miR-124 on the protein expression of CAPN2, western blot analysis was performed to detect the protein levels of CAPN2 in miR-124 mimic or mimic control-transfected cells. We observed a drop decrease in the protein expression of CAPN2 in the miR-124 mimic-transfected HT29 and SW480 cells as compared to their NC-transfected cells (Figure 4F).

### The Effect of miR-124 Overexpression on L-OHP-Based Chemotherapy in Nude Mice Bearing Xenograft Tumors

**Figure 5:**
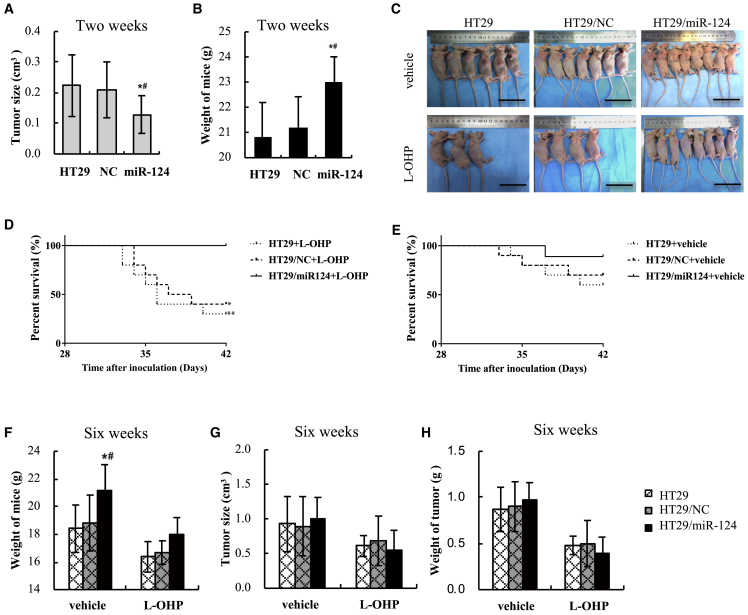
miR-124 Enhanced the Anti-Cancer Effect of Oxaliplatin-Based Chemotherapy in Mice Bearing HT29 Cells Stably Transfected with/without miR-124 or miR-NC (A and B) Tumor size (A) and weight of mice (B) were determined after 2 weeks of inoculation and analyzed with a histogram. (C) Anesthetized mice were photographed at the end of the experiment (scale bars, 5 cm). (D and E) The Kaplan-Meier plotter presented the 6-week overall survival of mice treated with L-OHP (D) or vehicle (E), respectively. ^∗∗^p < 0.01 and *p < 0.05, compared with HT29/miR124+L-OHP. (F) The weights of mice in each subgroup were evaluated at the end of the experiment. (G and H) The volumes (G) and weights (H) of subcutaneous xenografts in each subgroup were evaluated at the end of the experiment. Data are shown as means ± SEM of tumor measurements. ∗p < 0.05 versus control; ^#^p < 0.05 versus miR-NC.

Xenograft tumors of HT29 cell lines were established as shown in Figure S1. At the end of 2 weeks of inoculation, tumors from HT29 cells were statistically larger than those from HT29/miR-124 cells (0.21 ± 0.02 versus 0.12 ± 0.02 cm^3^, p = 0.001, Figure 5A). Alternatively, the weights of nude mice in the HT29 group were statistically lower than those in the HT29/miR-124 group (p < 0.001, Figure 5B). In addition, similar tumor sizes and weights of mice were observed between the HT29 group and the HT29/NC group. The results indicated that miR-124 overexpression inhibited tumorigenesis of HT29 cells, so as to inhibit tumor growth-induced weight loss of mice.

Figures 5C and 5D show that, in the three L-OHP-treated groups, univariate survival analysis showed that the group with high miR-124 expression was correlated with better OS than that in the low miR-124 expression group (HT29/miR124+L-OHP versus HT29+L-OHP, p = 0.004; HT29/miR-124+L-OHP versus HT29/NC+L-OHP, p = 0.010; HT29/NC+L-OHP versus HT29+L-OHP, p = 0.559, Figure 5D). Six-week OS was 100% (n = 8) in the HT29/miR124+L-OHP group, while it was only 30% (n = 10) in the HT29+L-OHP group and 40% (n = 10) in the HT29/NC+L-OHP group. In the three vehicle-treated groups, the 6-week OS of mice in the HT29/miR-124+vehicle group (89%, n = 9) was also higher than that in the HT29+vehicle group (60%, n = 10) and in the HT29/NC+vehicle group (70%, n = 10), although there were no statistically significant differences (HT29/miR124+vehicle versus HT29+vehicle, p = 0.156; HT29/miR124+vehicle versus HT29/NC+vehicle, p = 0.315, Figure 5E).

Six weeks after inoculation, in the three vehicle-treated groups, the weights of mice in the HT29/miR-124+vehicle group were still significantly higher than those in the HT29+vehicle group and HT29/NC+vehicle group (miR-124+vehicle versus HT29+vehicle, p = 0.018; miR-124+vehicle versus HT29/NC+vehicle, p = 0.042, Figure 5F). In the three L-OHP-treated groups, the weights of mice in the HT29/miR-124+L-OHP group were also higher than those in the HT29+L-OHP group and HT29/NC+L-OHP group (miR-124+L-OHP versus HT29+L-OHP, p = 0.088; miR-124+L-OHP versus HT29/NC+L-OHP, p = 0.100), although there was no statistical significance. These data indicated that miR-124 overexpression could protect nude mice from xenograft tumor-induced weight loss in conditions with or without chemotherapy. Moreover, the weights of nude mice that received L-OHP were all lower than those of nude mice that received vehicle, indicating that L-OHP induced weight loss of mice. This is consistent with the finding that CRC patients who receive L-OHP always lose weight in the clinic.

Interestingly, in the three L-OHP-treated groups, tumor sizes of the HT29/miR-124+L-OHP group were just slightly smaller than those of the HT29+L-OHP group and HT29/NC+L-OHP group (miR-124+L-OHP versus HT29+L-OHP, p = 0.761; miR-124+L-OHP versus HT29/NC+L-OHP, p = 0.504, Figure 5G). Meanwhile, in the three vehicle-treated groups, tumors in the HT29/miR-124+vehcile group grew more rapidly than did those in the HT29+vehicle group and HT29/NC+vehicle group at the end of chemotherapy treatment (Figure 5G). These data indicated that L-OHP delayed tumor development and the overexpression of miR-124 further improved the anti-tumor effect of L-OHP after 3 weeks of chemotherapy.

Furthermore, xenograft tumors were harvested and weighed after mice were sacrificed at the end of chemotherapy. In consistent with the results of Figure 5G, in the three vehicle-treated groups, weights of tumors in the HT29/miR124+vehicle group were slightly greater than those in the HT29+vehicle group and HT29/NC+vehicle group (miR-124+vehicle versus HT29+vehicle, p = 0.663; miR-124+vehicle versus HT29/NC+vehicle, p = 0.703). However, in the three L-OHP-treated groups, weights of tumors in the HT29/miR-124+L-OHP group were mildly lower than those in the HT29+L-OHP group and HT29/NC+L-OHP group (miR-124+L-OHP versus HT29+L-OHP, p = 0.775; miR-124+L-OHP versus HT29/NC+L-OHP, p = 0.532, Figure 5H).

**Figure 6:**
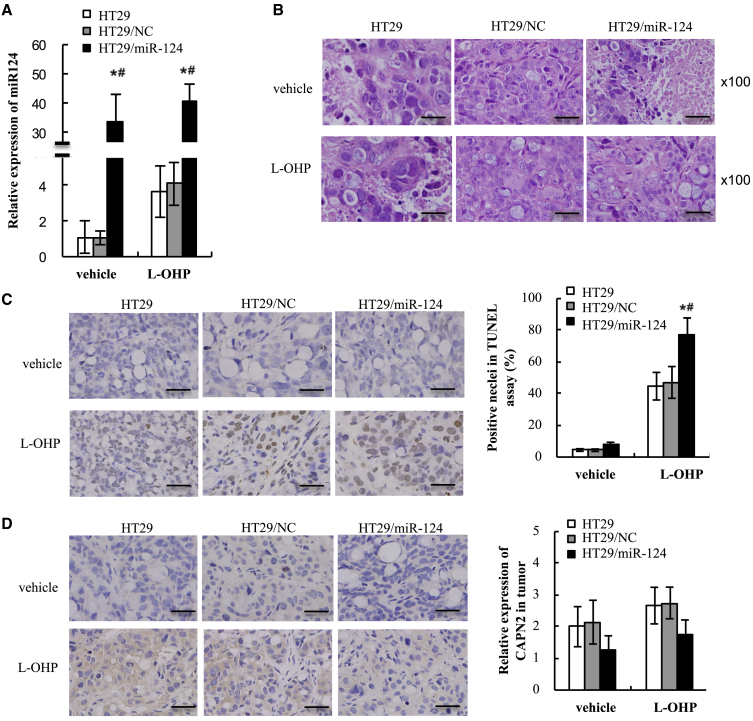
miR-124 Increased Apoptosis and Suppressed CAPN2 Protein Expression in Xenografts of Mice That Received Oxaliplatin-Based Chemotherapy (A) miR-124 expression in harvested xenografts was determined by quantitative real-time PCR. (B) Tumor morphology was analyzed by H&E staining. (C) TUNEL-positive cells were examined in xenografts by IHC staining and analyzed with a histogram. (D) Protein expression of CAPN2 in xenografts was determined by IHC staining and analyzed with a histogram. Original magnification, ×100. Scale bars, 50 μm. Data are presented as means ± SEM of measurements. ∗p < 0.05 versus control; ^#^p < 0.05 versus NC.

miR-124 promoted apoptosis and inhibited CAPN2 protein expression in xenograft tumors of CRC. After 6 weeks of inoculation, all mice were sacrificed and tumors were harvested, and miR-124 expression was validated by quantitative real-time PCR. As expected, miR-124 was still ectopically expressed in the tumors from HT29/miR-124 cells after 6 weeks of L-OHP or vehicle treatment (p < 0.001, Figure 6A). Furthermore, miR-124 expression levels in L-OHP-treated groups were higher than those in the corresponding vehicle treated groups (p < 0.001), suggesting that L-OHP may enhance miR-124 expression in CRC cells. Harvested tumors were stained with hematoxylin and eosin (H&E) and are shown in Figure 6B. In the three vehicle-treated groups, several large necrotic areas in the tumors were observed (Figure 6B). In the three L-OHP-treated groups, a few necrotic areas were found in the tumors. However, the size and number of necrotic areas in L-OHP-treated tumors were much lower than those in the three vehicle-treated groups.

The pro-apoptotic effect of L-OHP was evaluated by terminal deoxynucleotidyltransferase-mediated dUTP nick end labeling (TUNEL) staining in the tumors. As shown in Figure 6C, TUNEL-positive cells were markedly induced by L-OHP treatment and significantly enhanced by miR-124-overexpression (miR-124+L-OHP versus HT29+L-OHP, p = 0.016; miR-124+L-OHP versus HT29/NC+L-OHP, p = 0.025, Figure 6C). In order to determine the role of CAPN2 in miR-124-regulated chemosensitivity to L-OHP, CAPN2 protein expression in the tumors was evaluated by immunohistochemistry (IHC) staining. Figure 6D showed that CAPN2 protein expression levels in the HT29/miR-124+vehicle group were markedly reduced compared to the HT29+vehicle group and HT29/NC+vehicle group (miR-124+vehicle versus HT29+vehicle, p = 0.024; miR-124+vehicle versus HT29/NC+vehicle, p = 0.022, Figure 6D). The CAPN2 protein expression levels in the HT29/miR-124+L-OHP group also significantly decreased compared to the HT29+L-OHP group and HT29/NC+L-OHP group (miR-124+L-OHP versus HT29+L-OHP, p = 0.011; miR-124+L-OHP versus HT29/NC+L-OHP, p = 0.006).

## Discussion

To the best of our knowledge, this is the first study to demonstrate the relationship between miR-124 expression and the chemotherapy of CRC patients. We found that low miR-124 expression was significantly correlated with worse OS in CRC patients who received FOLFOX or XELOX. The main active ingredients of both FOLFOX and XELOX are L-OHP and 5-FU. Then, we observed that miR-124 overexpression enhanced chemosensitivity of CRC cells to L-OHP, but not to 5-FU, through enhancing L-OHP-induced cytotoxicity and apoptosis. Furthermore, miR-124 overexpression increased OS of mice injected with CRC cells and that received L-OHP-based chemotherapy. All of these data indicated that miR-124 could enhance the anti-cancer effect of L-OHP-based chemotherapy in CRC.

Note that L-OHP-based chemotherapy only increased OS of mice bearing HT-29/miR-124 cells (100% versus 89%). The 6-week survival rates of mice in the HT29+L-OHP group and HT29/NC+L-OHP group were lower than those in the HT29+vehicle group and HT29/NC+vehicle group, respectively (30% versus 60%, 40% versus 70%). This might be attributed to the side effect of L-OHP. It provides a hint that L-OHP-based chemotherapy should be preferentially applied to CRC patients with high expression levels of miR-124. It also tells us that chemotherapy does not improve survival of all patients. In some cases, chemotherapy might shorten the survival period because of its side effects. Therefore, identification of prognosis markers for different kinds of chemotherapies are very important in anti-cancer therapy.

Alternatively, miR-124 inhibited tumorigenesis and increased OS of mice bearing CRC cells even in the condition without chemotherapy. At the end of 2 weeks of inoculation, the volumes of tumors from HT29/miR-124 cells were statistically smaller than those from HT29 and HT29/NC cells. The weights of mice bearing HT29/miR-124 cells were higher than those from HT29 and HT29/NC cells. After 6 weeks of inoculation, the weights of mice in the HT29/miR-124+vehicle group were still markedly higher than those in the HT29+vehicle group and HT29/NC+vehicle group. Interestingly, the average tumor size in the HT29/miR-124+vehicle group was slightly higher than those in the HT29 and HT29/NC groups. We observed that the mice in the HT29+vehicle group and HT29/NC+vehicle group were thin and weak, and their xenograft tumors grew slowly in the second half of the experiment. Furthermore, the 6-week survival rate of the HT29/miR-124+vehicle group (89%) was higher than those of the HT29+vehicle group (30%) and HT29/NC+vehicle group (40%). It is consistent with our previous published finding that downregulated miR-124 expression was associated with worse prognosis in CRC patients.^14^

We also found that miR-124 expression levels increased in xenograft tumors after L-OHP treatment compared with those after vehicle treatment. Hayward et al.^22^ reported that L-OHP increased p53 and Bax expression to induce apoptosis in CRC cells. Furthermore, Liu et al.^23^ reported that p53 could bind to the promoter of miR-124 to promote its expression. Therefore, we supposed that L-OHP increased p53 expression levels and thereby upregulated miR-124 expression. L-OHP promoted miR-124 expression, suggesting that L-OHP may elicit its function through miR-124. Therefore, miR-124 will facilitate L-OHP to kill tumor cells when it is exotically introduced into the tumor cells, and tumor cells with higher levels of miR-124 are more sensitive to L-OHP.

Interestingly, studies have revealed that the CAPN protein systems are dysregulated in certain cancers. For example, the endogenous inhibitor of CAPN protein, calpastatin, is downregulated in nasopharyngeal carcinoma.^24^ In our present study, miR-124 overexpression suppressed CAPN2 protein expression by directly targeting the 3′ UTR of CAPN2 in CRC cells. Similarly, Storr et al.^21^ reported that CAPN2 protein expression is associated with response to platinum based chemotherapy, progression-free survival and OS in ovarian cancer. Furthermore, Lakshmikuttyamma et al.^19^ have reported that CAPN2 protein was overexpressed in human colorectal adenocarcinomas. Overall, our results suggest that miR-124-induced CAPN2 protein degradation mediates increased chemosensitivity to L-OHP in CRC cells and xenograft tumors. Thus far we have not been able to identify CAPN2 targets that might explain its role in the regulation of cell apoptosis and chemosensitivity. However, based on the observations shown in the present study and previous studies reported by others, we may suggest that inhibition of CAPN2 might sensitize cancer cells to the killing effects of some therapeutic drugs.

In conclusion, miR-124 was a significant predictor of OS and a prognostic factor for L-OHP-based chemotherapy in CRC patients. Further mechanistic studies are warranted to dissect the complex pathways carefully for miR-124 in chemotherapy sensitivity. Along with further research into its targets and upstream regulators, miR-124 could be considered as both a predictor of L-OHP-based chemotherapy for personalized treatment and a therapeutic target for CRC in the clinic.

## Materials and Methods

### Patients

All patients included in the study underwent surgical resection procedures at the Gastrointestinal Surgery Department, Affiliated West China Hospital of Sichuan University, Chengdu, China, from July 2009 to December 2012. The eligible cases were primary CRC patients (stage II or III) who received postoperative chemotherapy with FOLFOX or XELOX, without preoperative radiotherapy or chemotherapy. Patients with hereditary syndromes (e.g., familial adenomatous polyposis [FAP], hereditary nonpolyposis CRC [HNPCC]) or inflammatory bowel syndromes were excluded. The patients consisted of 138 men and 82 women with a median age of 66 years (range, 33–75 years). The surgical specimens included primary CRC together with the matched normal mucosa adjacent to the proximal excision margin. All specimens were snap-frozen immediately in liquid nitrogen and stored at −80°C until analysis.

The specimens were examined for the presence of tumor cells, which usually were at least around 80%, as well as normal mucosa, at the Department of Pathology in the West China Hospital. The grade of differentiation was graded as well, as moderately or poorly differentiated. As a regular follow-up program for the patients, a combination of physical examination (including digital examination), blood chemistry test (including measurement of carcinoembryonic antigen levels), chest X-ray, surveillance colonoscopy, and abdominal computed tomography (CT) was performed. At the end of March 2018, the mean follow-up period was 5.77 years. The study was approved by the Sichuan University Ethics Committee, and written informed consent was obtained from all the participants.

### Cell Lines

HEK293T cells and four human CRC cell lines (SW620, HCT116, HT29, and SW480) were purchased from the Shanghai Institute of Biochemistry and Cell Biology, Chinese Academy of Sciences (Shanghai, China). The cells were cultured in Dulbecco’s modified Eagle’s medium (DMEM, Gibco-BRL, Grand Island, NY, USA) supplemented with 10% fetal bovine serum (BD Biosciences, Bedford, MA, USA), 100 U/mL penicillin sodium and 100 μg/mL streptomycin sulfate (Beyotime Biotechnology, China). FHC normal cells were a gift from Linköping University. The FHC cells were cultured in a 1:1 mixture of Ham’s F12 and DMEM containing *N*-2-hydroxyethylpiperazine-*N*′-2-ethanesulfonic acid (HEPES) (25 mM), cholera toxin (10 ng/mL), insulin (5 μg/mL), transferrin (5 μg/mL), and hydrocortisone (100 ng/mL) (Sigma-Aldrich, China) and supplemented with 10% FCS. The cultures were maintained at 37°C in 5% CO_2_ and 95% humidity.

### Xenograft Tumors in Nude Mice

To investigate the effects of miR-124 on chemosensitivity *in vivo*, a xenograft animal model was used. Sixty male BALB/c nude mice (7 weeks old) were purchased from the Laboratory Animal Center of Chengdu Dashuo Biological Technology (Chengdu, China) and bred in specific pathogen-free (SPF) conditions. Mice were dispatched into three groups (20 mice/group) and respectively inoculated with HT29, HT29/NC, or HT29/miR-124 cells. Treatment schedules and grouping strategies were performed as shown in Figure S2. Briefly, 2.5 × 10^6^ HT29, HT29/NC, or HT29/miR-124 cells were suspended in 200 μL of cell culture medium and injected subcutaneously into the back next to the right forelimb of mice. Two weeks later, each group of mice bearing xenograft tumors was further equally divided into two sub-groups and started receiving an intraperitoneal (i.p.) injection of 400 μL of L-OHP (6 mg/kg) or vehicle (5% glucose) twice a week (on Monday and Thursday) for 3 weeks. Therefore, there were six sub-groups of nude mice in total and named as HT29+vehicle, HT29/NC+vehicle, HT29/miR-124+vehicle, HT29+L-OHP, HT29/NC+L-OHP, and HT29/miR-124+L-OHP, respectively. Tumor size was measured every week and calculated using the following formula: 0.5(length × width^2^). Six weeks after inoculation, all survival nude mice were euthanized by injecting a large dose of chloral hydrate, after which xenograft tumors were dissected. All animal studies were conducted in the Animal Institute of Sichuan University according to the protocols approved by the Medical Experimental Animal Care Commission of the University.

### Preparation of Chemical Reagents

Both 5-FU (F6627) and L-OHP (O9512) were purchased from Sigma-Aldrich. A stock solution of 5-FU (200 mM) was prepared by dissolving 5-FU powder into DMSO and diluting with cell culture medium to required concentrations before use. *In vitro*, a stock solution of L-OHP (10 mM) was prepared by dissolving L-OHP into phosphate-buffered saline (PBS) and diluted with culture medium to required concentrations before use. *In vivo*, 30 mg of L-OHP was dissolved into 100 mL of 5% glucose injection (used as vehicle) and filtered with a 0.22-μm filter membrane.

### Lentiviral Packaging and Stable Cell Line Establishment

Lentiviruses carrying miR-124 or miR-NC were packaged using a lentiviral packaging kit in HT29 or SW480 cells following the manufacturer’s instructions (LAND, Guangzhou, China). HT29/miR-124 and SW480/miR124 cells were established in our laboratory from HT29 and SW480 cells, respectively, by transfecting with pLVX-IRES-ZsGreen1+miR-124 viruses. HT29/NC and SW480/NC cells, used as NC cell lines, were obtained from HT29 and SW480 cells by transfecting with pLVX-IRES-ZsGreen1+control viruses. The lentiviral vector (pLVX-IRES-ZsGreen1 viruses) carrying miR-124 or miR-NC has a green fluorescent protein (GFP) tag, which can be used to check the efficiency of package delivery using a microscope. HT29 and SW480 cells were transduced by lentiviral soup and selected by puromycin to establish stable cell lines.

### Cytotoxic Assay

The cells in the logarithmic phase of growth were seeded on 96-well plates at a density of 2000-3000 cells/well overnight and then were subjected to various concentrations of 5-FU or L-OHP for indicated periods. Cell viability was assayed by CCK-8 (Dojindo, Kumamoto, Japan) according to the manufacturer’s instructions. Each assay was performed in triplicate.

### Flow Cytometry Analysis

The cells, treated with or without L-OHP, were harvested and washed twice with cold PBS and re-suspended in binding buffer containing annexin V-APC (BD Biosciences, Bedford, MA, USA) and subjected to 7-AAD staining incubation for 15 min at 4°C in the dark. The stained cells were analyzed with flow cytometry within 60 min. The percentage of apoptotic cells was calculated using FlowJo software (Verity Software House). Early-stage apoptosis is annexin V-positive and 7-AAD-negative (annexin V-APC^+^/7-AAD^−^), whereas late-stage apoptosis is annexin V/7-AAD-double positive (annexin V-APC^+^/7-AAD^+^).

### Isolation of RNA, Quantitative Real-Time PCR

Total RNA was extracted from cultured cells or nude mice tissue using a miRNeasy mini kit (217004, QIAGEN, Germany), and the cDNA was synthesized using the miScript II RT Kit (218161, QIAGEN, Germany) following the manufacturer’s instructions. Quantitative real-time PCR was performed in triplicate using a miScript SYBR Green PCR kit (218073, QIAGEN, Germany) on a quantitative real-time PCR system (iCycler iQ, Bio-Rad, Hercules, CA, USA). The primers for real-time PCR were as follows: Hs_-_miR-124a_-_1 miScript primer assay (218300, QIAGEN), Hs_-_RNU6-2_-_11 miScript primer assay (218300, QIAGEN). The expression level of miR-124 was determined relative to U6 as an internal control, and the fold increase was calculated by using the equation 2^−ΔΔCT^, where ΔCT = CT_miR-124_ − CT_U6_, and ΔΔCT = ΔCT_in CRC-miR-124 or -NC cells_ − ΔCT_in CRC cells_, or ΔΔCT = ΔCT_in tumor_ − ΔCT_in adjacent mucosa_. Primer sequences (QIAGEN) are available upon request.

### Qualitation and Quantitation of Quantitative Real-Time PCR Products

*In vitro*, the quantitative real-time PCR products were subjected to electrophoresis on a 4% agarose gel, to which ethidium bromide (10 mg/mL) had been added. Electrophoresis was carried out in Tris-borate-EDTA (TBE) buffer at 120 V for 30 min. The bands were visualized under UV illumination, and a densitometer (Bio-Rad) was used to scan the intensity of the bands.

### Western Blotting

Total protein was extracted in radioimmunoprecipitation assay (RIPA) lysis buffer, separated by SDS-PAGE, and then transferred onto a polyvinylidene fluoride (PVDF) membrane (Millipore, Bedford, MA, USA). The membranes were blocked in Tris-buffered saline with Tween 20 (TBST) containing 5% dry skim milk at 37°C for 2 h and incubated with the primary antibodies at 4°C overnight against pro-caspase-3 (1:500), active caspase-3 (1:500), CAPN2 (1:1,000), or β-actin (1:2,500), followed by an incubation with horseradish peroxidase (HRP)-conjugated secondary antibody (1:5,000) for 1 h at 37°C. The primary antibodies of pro-caspase-3, active caspase-3, and β-actin and the secondary antibody were from Cell Signaling Technology (Beverly, MA, USA). The primary antibody of CAPN2 was from Abcam (Cambridge, UK). Employing an enhanced chemiluminescence kit (Millipore), the bands were automatically visualized using a ChemiDoc XRS+ system (Bio-Rad, Hercules, CA, USA) and quantitatively analyzed with Image Lab software (Bio-Rad). β-Actin expression was used as the internal control to normalize the sample loading amounts.

### Luciferase Assays

The WT 3′ UTR segment of CAPN2 was cloned downstream of the luciferase coding region in the psiCHECK vectors (Promega, Madison, WI, USA) to generate luciferase reporter vector CAPN2 3′ UTR-WT. The corresponding mutant constructs (CAPN2 3′ UTR-mut) were created by mutating the seed regions of the miR-124 binding sites. Cells were plated into 24-well plates until 80% confluence before transfection. The WT or mutant CAPN2 3′ UTR plasmid was transiently co-transfected with miR-124 mimics or NC into HEK293T cells. Cell lysates were harvested 24 h post-transfection, and luciferase activities were measured by the Dual-Luciferase reporter assay system (Promega). Luciferase activity was normalized by Renilla luciferase activity.

### IHC and H&E Staining

The harvested xenograft tumors were fixed in 10% formalin, dehydrated, and embedded in paraffin. Then, 5-μm-thick sections were stained against CAPN2 protein (1:700, ab713, Abcam). Positive cells were visually identified in 10 randomly selected fields and imaged at a magnification of ×100. Formalin-fixed and paraffin-embedded 3-mm tissue sections of each mouse’s xenograft tumor were stained using the H&E technique. The slides were analyzed by a pathologist.

### TUNEL Staining

Sections were fixed in 4% paraformaldehyde (PFA) for 15 min at room temperature, rinsed in distilled PBS, and incubated with equilibration buffer for 1 min. A TUNEL assay was subsequently performed by using an ApopTag peroxidase *in situ* apoptosis detection kit (Merck Millipore, Temecula, CA, USA) in accordance with the manufacturer’s instructions. Apoptotic cells were visually identified in 10 randomly selected fields and imaged at a magnification of ×100. Apoptotic cells were counted to calculate the percentage of TUNEL-positive cells.

### Statistical Analysis

Data were represented as means ± SD from at least three independent experiments except those specifically indicated. We compared two groups using the t test for continuous variables and a χ^2^ test for categorical variables. For survival analyses, we used the Kaplan-Meier method to compare OS differences between “high” and “low” expression groups and calculated the p value using the log-rank test in the survival package in GraphPad Prism 7.0. We used the Cox regression model to do the multivariable survival analysis, and Cox regression coefficients to generate nomograms. p < 0.05 was considered to be statistically significant. Statistical analysis was performed using SPSS software (version 17.0, SPSS, Chicago, IL, USA). All data generated or analyzed during this study are included in this published article or the Supplemental Information.

## Author Contributions

X.-Q.X. conducted the most *in vitro* and *in vivo* experiments. M.-J.W. and J.P. collected and analyzed specimens of CRC patients. L.-P.L. and N.W. helped with the animal experiment. K.-L.C. helped with cell culture. Z.-Y.L. helped with H&E and IHC staining. B.Z. helped with quantitative real-time PCR. X.-Q.X., M.-J.W., Y.L., Z.-G.Z., and X.-F.S. designed this research and prepared the manuscript. All authors read and approved the final manuscript.

## Conflicts of Interest

The authors declare no competing interests.

## Supplemental Information

Document S1. Figures S1 and S2

Document S2. Article plus Supplemental Information
