# Supplementary material for: miR-124 Intensified Oxaliplatin-Based Chemotherapy by Targeting CAPN2 in Colorectal Cancer
Source: Mol Ther Oncolytics. 2020 Apr 14;17:320–31. doi: 10.1016/j.omto.2020.04.003 (PMC7200624; doi:10.1016/j.omto.2020.04.003)
Supplement: Document S1. Figures S1 and S2 [file mmc1.pdf]

OMTO, Volume *17*

Supplemental Information

miR-124 Intensified Oxaliplatin-Based Chemotherapy by Targeting CAPN2 in Colorectal Cancer

Xu-Qin Xie, Mo-Jin Wang, Yuan Li, Lin-Ping Lei, Ning Wang, Zhao-Ying Lv, Ke-Ling Chen, Bin Zhou, Jie Ping, Zong-Guang Zhou, and Xiao-Feng Sun

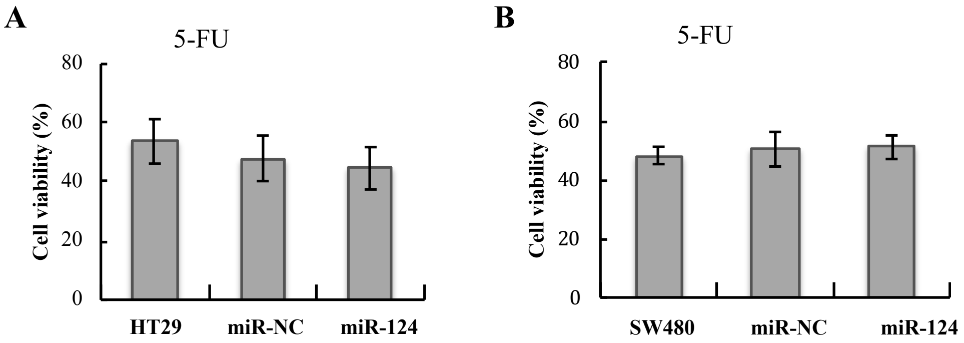

Fig. S1. miR-124 restoration didn't affect 5-FU-induced cytotoxicity in HT29 and SW480 cells. **A**, **B** Cytotoxic effect of 5-FU on HT29 cells (A) or SW480 cells (B) stably transfected with/without miR-124 or miR-NC were determined by CCK8 assay.

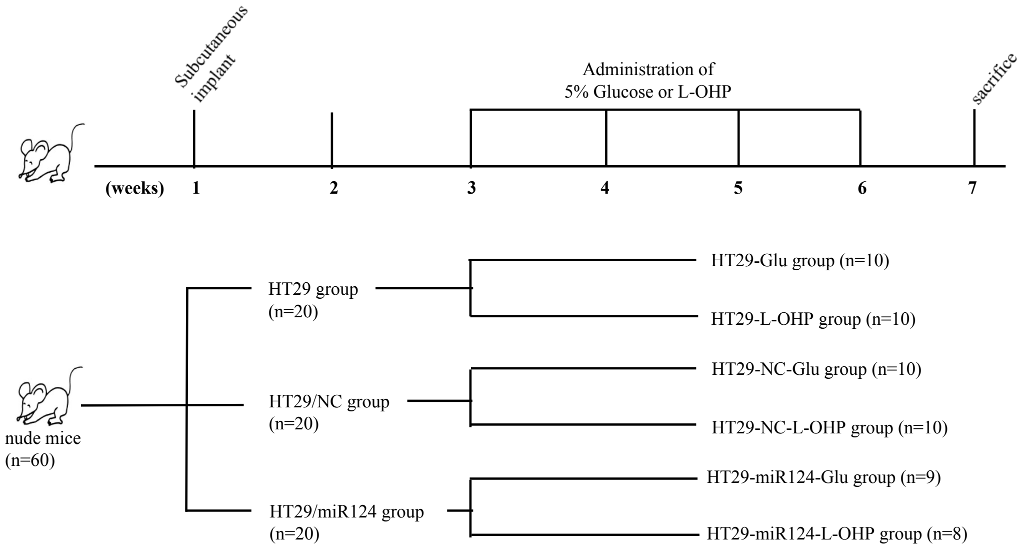

Fig. S2. The treatment schedule and grouping strategies were used to establish the xenograft model in nude mice. On the first day (Monday) of 1st week, sixty male BALB/c nude mice were dispatched into 3 groups (20 mice/group) and respectively inoculated with HT29 or HT29/NC or HT29/miR-124 cells. On the first day of 3rd week, each group of mice bearing xenograft tumors were further equally divided into 2 sub-groups. Thus, there were 6 sub-groups of nude mice in total and respectively named as HT29+vehicle, HT29/NC+vehicle, HT29/miR-124+vehicle, HT29+L-OHP, HT29/NC+L-OHP and HT29/miR-124+L-OHP. Then these mice started receiving an intraperitoneal injection (i.p.) of 400 μL L-OHP (6 mg/kg) or vehicle (5% Glucose) twice a week (on Monday and Thursday) for 3 weeks. Tumor size was measured every week and calculated using the following formula: $0.5 \times \text{length} \times \text{width}^2$. Six weeks after inoculation, all survival nude mice received euthanasia by injecting large dose of chloral hydrate and xenograft tumors were dissected.
